# Immunity related genes in dipterans share common enrichment of AT-rich motifs in their 5' regulatory regions that are potentially involved in nucleosome formation

**DOI:** 10.1186/1471-2164-9-326

**Published:** 2008-07-09

**Authors:** Jesus Hernandez-Romano, Francisco J Carlos-Rivera, Heladia Salgado, Hector Lamadrid-Figueroa, Veronica Valverde-Garduño, Mario H Rodriguez, Jesus Martinez-Barnetche

**Affiliations:** 1 Centro de Investigación sobre Enfermedades Infecciosas, Instituto Nacional de Salud Pública, Av. Universidad 655, Col Sta Maria Ahuacatitlan, CP 62508, Cuernavaca, Morelos, México; 2 Independent Consultant on Computational Sciences, Cuernavaca, Morelos, México; 3 Centro de Ciencias Genómicas, Universidad Nacional Autónoma de México, Av Universidad s/n Col Chamilpa 62210, Cuernavaca, Morelos, México; 4 Dirección de Estadística, Centro de Investigación en Evaluación y Encuestas (CIEE), Instituto Nacional de Salud Pública, Av Universidad 655, Col Sta Maria Ahuacatitlan, CP 62508, Cuernavaca, Morelos, México; 5 Ingeniería en Biotecnología, Universidad Politécnica del Estado de Morelos, Boulevard Cuauhnahuac 566, Col Lomas del Texcal, Jiutepec, Morelos, México

## Abstract

**Background:**

Understanding the transcriptional regulation mechanisms in response to environmental challenges is of fundamental importance in biology. Transcription factors associated to response elements and the chromatin structure had proven to play important roles in gene expression regulation. We have analyzed promoter regions of dipteran genes induced in response to immune challenge, in search for particular sequence patterns involved in their transcriptional regulation.

**Results:**

5' upstream regions of *D. melanogaster *and *A. gambiae *immunity-induced genes and their corresponding orthologous genes in 11 non-melanogaster drosophilid species and *Ae. aegypti *share enrichment in AT-rich short motifs. AT-rich motifs are associated with nucleosome formation as predicted by two different algorithms. In *A. gambiae *and *D. melanogaster*, many immunity genes 5' upstream sequences also showed NFκB response elements, located within 500 bp from the transcription start site. In *A. gambiae*, the frequency of ATAA motif near the NFκB response elements was increased, suggesting a functional link between nucleosome formation/remodelling and NFκB regulation of transcription.

**Conclusion:**

AT-rich motif enrichment in 5' upstream sequences in *A. gambiae, Ae. aegypti *and the *Drosophila *genus immunity genes suggests a particular pattern of nucleosome formation/chromatin organization. The co-occurrence of such motifs with the NFκB response elements suggests that these sequence signatures may be functionally involved in transcriptional activation during dipteran immune response. AT-rich motif enrichment in regulatory regions in this group of co-regulated genes could represent an evolutionary constrained signature in dipterans and perhaps other distantly species.

## Background

Organismal complexity is dependent on the network that regulates gene expression, rather than the number of genes in its genome [1-3]. Thus, one of the biggest challenges in postgenomic research is understand the regulatory mechanisms controlling location, timing and intensity of gene expression.

Organisms are permanently sensing changes in their environment. Environmental agents activate cellular signaling pathways that lead to a rapid expression of specific genes to respond to changes. These pathways transmit their signal to specific transcription factors (TFs) which gain access to response elements (REs) located in promoter and enhancer regions of the corresponding gene [2] resulting in transcriptional activation. In eukaryotes these protein-DNA interactions occur in the context of a chromatin template within the cell nucleus. The fundamental unit of chromatin is the nucleosome, composed by a segment of 146 base pairs of double stranded DNA wrapped around a core of histone proteins [4]. Initially, nucleosomes were regarded as structures required for the packing of long DNA molecules into the cellular nucleus [5], but it is now clear that chromatin structure plays a central role in the regulation of gene expression [6-9]. At least three mechanisms have been proposed for the active role of chromatin in transcriptional regulation. First, by preventing TF binding to its cognate RE as revealed by the pioneering studies in the expression of PHO5 gene in response to phosphate starvation [10]. Secondly, wrapping DNA in nucleosomes may promote transcription by allowing closely adjacent RE access to their cognate TF [11,12]. Third, nucleosomes may approximate distant regulatory elements, as it occurs in the alcohol-dehydrogenase (Adh) promoter region of *Drosophila *[13].

Nucleosomes are located in preferred positions with respect to DNA sequence [14-21]. It has been shown that on a statistical level, groups of experimentally obtained nucleosomal sequences display periodicity in the occurrence of dinucleotides such as GG, TA, TG, and TT [14,15,20] or trinucleotides such as VWG ([G/C/A] [A/T]G) [19]. This periodicity tends to occur approximately every 10 bp, coinciding with one turn of the DNA chain and confers better bending properties required for wrapping DNA around the histone core. However, this periodicity is difficult to identify on individual nucleosomal sequences due to a low signal/noise ratio. The non-random distribution of nucleosomes suggests that some DNA sequences are more likely to form stable nucleosomes, and therefore nucleosome forming sequences could be predicted using computational methods based on the sequence features identified so far [20,22].

Immune responses are inducible phenomena resulting from a close relationship between the environment, pathogen signal detection systems and the gene expression machinery [23]. Upon pathogen recognition, several transduction pathways are activated leading to the activation of TFs that induce gene expression [24]. In *Drosophila melanogaster*, the Toll and Imd pathways converge in the activation of the NFκB/Rel-related TFs, Dif and Relish, respectively, which bind to NFκB REs located in the 5' upstream regions of antimicrobial peptide genes, thus promoting their transcription [25].

Understanding the transcriptional regulation mechanisms during insect immune response is of fundamental interest in biology, but also could provide the rational basis for developing strategies to control vector borne diseases. In this work, we describe that immunity genes induced upon immune challenge in *D. melanogaster *and *Anopheles gambiae*, the main African malaria vector, share an enrichment of AT-rich motifs in their 5' regulatory regions. Enrichment of AT-rich motifs was also observed in 10 additional non-melanogaster *Drosophila *species and *Aedes aegypti *immunity orthologs. These motifs are different to REs in terms of statistical frequency and length. Their occurrence correlates with predicted nucleosomal positions [18,20,22], suggesting that AT-rich motifs may be involved in chromatinization and transcriptional regulation of immunity related transcriptional gene modules in these insects.

## Results

### Regulatory regions of immunity-related co-expressed genes of *Anopheles gambiae *and *Drosophila melanogaster *induced upon immune challenge are enriched in AT-rich specific DNA motifs

We used public available and author provided microarray databases [26,27], coupled to bioinformatics analysis tools for regulatory sequences, to identify sequence patterns potentially involved in transcriptional regulation operating during immune response in *D. melanogaster *and *A. gambiae *.

**Figure 1:**
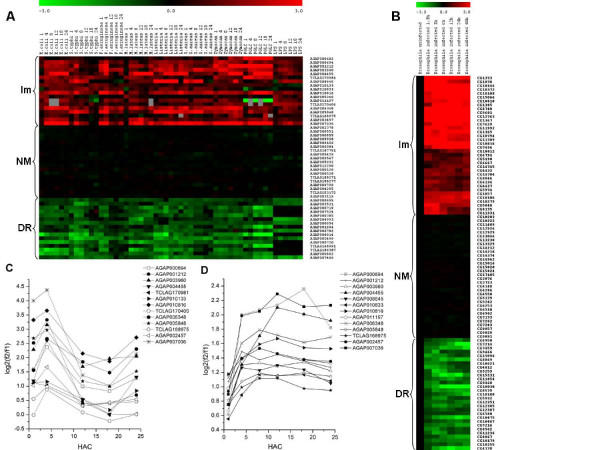
**Expression profiles of immunity, non-modified and repressed genes of *A. gambiae *(A) and *D. melanogaster *(B). In red color are shown the immunity (Im) induced genes, in black the non-modified (NM) genes and green the down-regulated (DR) genes.** The expression values are log_2_(f2/f1), as reported by Dimopoulos [26] and De Gregorio [27]. C) and D) are examples of co-expressed genes of *A. gambiae *in response to *P. aeruginosa *and *E. coli*, respectively. 4a-3B cells of *A. gambiae *were challenged with nine microbial stimuli and analyzed at 1, 4, 8, 12, 18 and 24 hours post-challenge (except for *P. aeruginosa*, for which the 8 h sample was omitted). For *D. melanogaster*, adult males were challenged by pricking the thorax with a needle dipped into a bacterial culture of *Escherichia coli *and *Micrococcus luteus *and analyzed at 1.5, 3, 6, 12 and 24 hours after challenge (HAC).

Microarray data describing the temporal transcriptional profile for 13,196 *D. melanogaster *genes [27], and 2,883 *A. gambiae *genes [26] in response to various immune challenges were used to select genes with the following expression profiles: immunity induced, repressed and non-modified (Figure 1). The *Drosophila *microarray data were obtained using Oregon^R ^adult males challenged with *Escherichia coli *and *Micrococcus luteus *[27]. The *Anopheles *microarray data were obtained using the *A. gambiae *cell line 4a-3B challenged with several bacteria species or microbial products [26]. Gene groups were selected according to 1) gene ontology and Interpro assignments as well as induction after an immune challenge, 2) genes that were not modified upon immune challenges, and 3) down-regulated genes upon immune challenges. Two additional groups were added as controls: 4) randomly selected genes and 5) computer-randomly generated sequences (artificial). Table 1 presents the number of analyzed genes in each group and Tables 2, 3, 4, 5, 6, 7 list the Ensembl or AnoEST ID, gene description, and chromosomal location for the genes used in the analysis. The expression profiles of the three gene groups (immunity induced, repressed and non-modified) of both species are shown in Figure 1.

**Table 1:** Number of 5' upstream sequences analyzed according expression pattern and functional class.

**Organism**	**Immunity up-regulated**	**Down-regulated**	**Non modified**	**Random genes**	**Artificial**
***Anopheles gambiae***	18	16	19	20	20
***Drosophila melanogaster***	36	29	32	30	30

Groups of *A. gambiae *and *D. melanogaster *genes selected for *in silico *analysis for searching potential DNA regulatory motifs. 2500 nucleotides were analyzed.

**Table 2:** Immunity-related genes from *A. gambiae*.

Gene ID*	**Description**	**Chromosome Name**	**Band**
AGAP000182	Serine protease domain (IPR001314)	X	4A
AGAP000694	Antimicrobial peptide Cecropin (IPR000875)	X	1C
AGAP001212	Peptidoglycan recognition protein long class (PGRP-LB)	2R	7A
AGAP002457	Chitin binding domain, Glycoside hydrolase (IPR002557, IPR001579)	2R	12B
AGAP003960	Serine protease domain, Gastrulation defective precursor (IPR001314, IPR001254)	2R	17A
AGAP004455	Gram negative binding protein subgroup B (GNBPB1)	2R	19A
AGAP005246	Serpin	2L	21E
AGAP005848	Fibrinogen domain (IPR002181)	2L	23A
AGAP006348	LRR Toll	2L	24B
AGAP007036	Leucine-rich repeat (IPR001611)	2L	26C
TCLAG170406	Cactus	3R	29C
TCLAG170981	Phosphotyrosine interaction region (IPR006020)	3R	30E
AGAP008645	Putative infection responsive (Gambicin)	3R	31A
TCLAG168975	Dopa Decarboxylase isoform 1	3R	33B
AGAP010133	Scavenger receptor class B Croquemort type	3R	37A
AGAP010816	Thioester-Containing Protein (TEP3)	3L	39C
AGAP010833	CLIP-domain serine protease subfamily B (CLIPB14) (IPR001254, IPR006604)	3L	39C
AGAP011197	Fibrinogen	3L	41D

*According to Ensembl [28] and AnoEST [67].

The Ensemble Gene ID or AnoEST cluster ID, description, chromosome and band location are showed. For genes without description, InterPro annotation is showed.

**Table 3:** Immunity-related genes from *D. melanogaster*.

**Ensembl Gene ID**	**Description**	**Chromosome Name**	**Band**
CG10146	Attacin-A precursor	2R	51C
CG10794	Diptericin B	2R	55F
CG10810	Drosomycin precursor	3L	63D
CG10812	drosomycin-5	3L	63D
CG10816	Drosocin precursor	2R	51C
CG11331	Serpin-27A CG11331-PA	2L	26F
CG11709	Peptidoglycan-recognition protein-SA precursor (Protein semmelweis)	X	10C
CG11992	Nuclear factor NF-kappa-B p110 subunit (Relish protein) (Rel-p110)	3R	85C
CG12763	Diptericin precursor	2R	55F
CG1365	Cecropin-A1/A2 precursor	3R	99E
CG1367	Cecropin-A1/A2 precursor	3R	99E
CG1373	Cecropin-C precursor	3R	99E
CG1385	Defensin precursor	2R	46D
CG14704	Peptidoglycan-recognition protein-LB precursor	3R	86E
CG14745	Peptidoglycan-recognition protein-SC2 precursor	2R	44E
CG15066	Immune-induced peptide 23 precursor (DIM-23)	2R	55C
CG18106	Immune-induced peptide 2 precursor (DIM-2)	2R	55C
CG18108	Immune-induced peptide 1 precursor (DIM-1)	2R	55C
CG18279	Immune-induced peptides precursor (DIM-10; DIM-12; DIM-13; DIM-24)	2R	50A
CG18372	Attacin-B precursor	2R	51C
CG1857	Necrotic	2R	42F
CG1878	Cecropin-B precursor	3R	993E
CG4432	Peptidoglycan-recognition protein-LC	3L	67B
CG4437	Peptidoglycan-recognition protein-LF (PGRP-like protein)	3L	67B
CG4740	Attacin-C precursor	2R	50A
CG5490	Protein toll precursor	3R	97D
CG5848	NF-kappa-B inhibitor cactus.	2L	35F
CG5974	Probable serine/threonine-protein kinase pelle	3R	97E
CG6134	Protein spaetzle precursor	3R	97E
CG6667	Embryonic polarity protein dorsal	2L	36C
CG6794	Dorsal-related immunity factor Dif	2L	36C
CG7496	Peptidoglycan-recognition protein-SD precursor.	3L	66A
CG7629	Attacin-D	3R	90B
CG8175	Metchnikowin precursor	2R	52A
CG8846	Thor CG8846-PA	2L	23F
CG9681	Peptidoglycan-recognition protein-SB1 precursor	3L	73C

**Table 4:** Non-modified genes from *A. gambiae*.

Gene ID*	**Description**	**Chromosome Name**	**Band**
AGAP000120	Cytoskeleton-associated proteins (CAP-Gly)	X	4B
AGAP000528	HMG-I and HMG-Y, DNA-binding Basic-leucine zipper (bZIP) transcription factor	X	2B
AGAP012290	Transporter	X	2B
AGAP000551	Dehydrogenase, E1 component, Transketolase, central region	X	2B
AGAP000855	No description	X	5A
AGAP002378	Fumarate lyase, Delta crystallin	2R	12B
AGAP003119	Translation initiation factor 4C (1A)	2R	14B
AGAP004295	DTW domain	2R	18D
AGAP005032	Calreticulin/calnexin	2L	21A
AGAP005429	WD-40 repeat	2L	22B
AGAP005558	Peptidase M16, C-terminal	2L	22C
AGAP006084	Antifreeze protein, type I, Ubiquitin system component Cue	2L	23C
AGAP006729	Domain of unknown function DUF1907	2L	25C
AGAP009156	BRICHOS	3R	33C
AGAP009547	Endoplasmic reticulum targeting sequence, Torsin	3R	34C
TCLAG188271	Neutral zinc metallopeptidases, zinc-binding region signature	X	1D
TCLAG158277	Leucine aminopeptidase-related (PTHR11963)	2R	15D
TCLAG153172	RNA 3'-terminal phosphate cyclase, insert region	2L	20C
TCLAG187751	Malic oxidoreductase	X	4A

*According to Ensembl [28] and AnoEST [67].

**Table 5:** Non-modified genes from *D. melanogaster*

**Ensembl Gene ID**	**Description**	**Chromosome Name**	**Band**
CG10062	Peptidase M28	2R	56D
CG10203	Eggshell protein, Zinc finger, CCHC-type, RNA-binding region RNP-1 (RNA recognition motif)	2L	27C
CG11489	Serine/threonine protein kinase, active site	3L	79D
CG12929	No description	2R	45F
CG13046	No description	3L	72D
CG13230	No description	2R	47D
CG13329	Histone H3, Dopamine D4 receptor	2R	50A
CG14212	HAD-superfamily subfamily IB hydrolase, hypothetical 1	X	18D
CG14218	Vinculin/alpha-catenin	X	18D
CG14332	No description	3R	90A
CG14962	Zinc finger, C2H2-type	3L	63B
CG15016	Probable mitochondrial 28S ribosomal protein S6	3L	64B
CG15458	Putative 60S ribosomal protein L33	X	19D
CG15526	No description	3R	99D
CG15824	No description	2L	21E
CG17105	Eggshell protein	2L	32A
CG2076	BAX inhibitor related (PTHR23291)	X	10A
CG3723	Peptidase, cysteine peptidase active site, Dynein heavy chain, N-terminal, ATPase associated with various cellular activities	3R	93E
CG4148	Zinc finger, C2H2-type, HMG-I and HMG-Y, DNA-binding	2L	35D
CG4550	Opsin, KiSS-1 peptide receptor	3R	92B
CG4733	Calcium-binding EF-hand, Antifreeze protein, type I	3R	92B
CG5179	Cyclin-dependent kinase 9	2R	58F
CG5242	mitochondrial ribosomal protein L40	3R	86E
CG5675	Aminoacyl-tRNA synthetase, class I, Phosphotyrosine interaction region	X	16B
CG6253	60S ribosomal protein L14	3L	66D
CG6745	tRNA pseudouridine synthase D, TruD	3L	66D
CG6982	Claudin tight junction protein, Voltage-dependent calcium channel gamma	3R	94C
CG7173	Protease inhibitor, Kazal-type	3L	78D
CG7283	60S ribosomal protein L10a-2	3L	68E
CG9029	Orphan nuclear receptor, HMR type,	2L	26A
CG9091	Probable 60S ribosomal protein L37-A	X	13B
CG9961	Phosphoglycerate kinase	2L	23A

**Table 6:** Down-regulated genes from *A. gambiae*.

Gene ID*	**Description**	**Chromosome Name**	**Band**
AGAP000385	no description	X	2C
AGAP000654	Ribosomal protein S30, ubiquitin	X	1C
AGAP000655	Ribosomal protein S11	X	1C
AGAP000719	Adenosylhomocysteinase	X	1A
AGAP000862	Glycoside hydrolase	X	5A
AGAP001604	Putative Tyr/Ser/Thr phosphatase	2R	8D
AGAP002499	Probable methylmalonate-semialdehyde dehydrogenase, Mitochondrial precursor	2R	12C
AGAP003521	Alpha-2-macroglobulin RAP, C-terminal	2R	15D
AGAP004993	EGF-like, laminin	2L	20D
AGAP006782	ADP, ATP carrier protein 1 (ADP/ATP translocase 1)	2L	25D
AGAP007406	Elongation factor 1 alpha	2L	27D
AGAP007524	ST7	2L	28A
AGAP008914	no description	3R	32C
TCLAG186387	no description	X	3D
AGAP000720	no description	X	1A
TCLAG168991	no description	3R	33B

*According to Ensembl [28] and AnoEST [67].

**Table 7:** Down-regulated genes from *D. melanogaster*.

**Ensembl Gene ID**	**Description**	**Chromosome Name**	**Band**
CG10467	Aldose 1-epimerase	3L	65A
CG10475	Peptidase S1 and S6, chymotrypsin	3L	65A
CG10621	Homocysteine S-methyltransferase	2L	37B
CG11236	D-amino acid oxidase	2L	27B
CG11854	Hormone binding, cysteine peptidase active site	3R	96C
CG12351	Trypsin delta/gamma precursor	2R	47F
CG12385	Trypsin theta precursor	2R	47F
CG12387	Trypsin zeta precursor	2R	47F
CG15096	Sugar transporter superfamily	2R	55F
CG15231	Immune-induced peptide 4 precursor (DIM-4)	2R	57B
CG18030	Peptidase S1 and S6, chymotrypsin/Hap	3R	99F
CG18179	Peptidase S1 and S6, chymotrypsin/Hap	3L	67C
CG18180	Peptidase S1 and S6, chymotrypsin/Hap	3L	67C
CG18255	Stretchin-Mlck, isoform E	2R	52D
CG4178	Larval serum protein 1 beta chain precursor (Hexamerin 1 beta)	2L	21E
CG4812	Peptidase S1 and S6, chymotrypsin/Hap	2R	50A
CG4950	Leucine-rich repeat	3L	72D
CG5932	Esterase/lipase/thioesterase	3L	77C
CG6580	Peptidase S1 and S6, chymotrypsin/Hap	3L	65A
CG7214	No description	2L	28C
CG7216	Adult cuticle protein 1 precursor (dACP-1)	2L	28C
CG7459	Copper transporter 1B	3R	84F
CG8562	Peptidase M14, carboxypeptidase A	3L	65F
CG8579	Peptidase S1 and S6, chymotrypsin/Hap	2R	44E
CG8867	Peptidase S1 and S6, chymotrypsin/Hap	2L	25B
CG8869	Peptidase S1 and S6, chymotrypsin/Hap	2L	25B
CG9259	No description	2L	39A
CG9466	Glycoside hydrolase, family 38	2L	29F
CG9468	Glycoside hydrolase, family 38	2L	29F

To investigate whether the 5' regulatory regions of immunity-related genes shared common DNA motifs, 2500 bp 5' upstream sequences (5'-US) were recovered using Biomart, of Ensembl [28] and analyzed for statistically overrepresented motifs of 2 to 8 nucleotides in length, using Oligo-Analysis, which is based on binomial distribution [29]. The background oligonucleotide frequencies were estimated calculating the relative frequencies of all possible oligonucleotides (ranging from 2 to 8 bp) within the 5'-US of 2500 bp of length of 13,166 *A. gambiae *or 13,172 *D. melanogaster *genes. Oligonucleotide occurrences were counted for each group of 5'-US and their statistical significance was estimated on the basis of the background frequencies. The significance index (sig_occ_) reflects the degree of overrepresentation of each motif on a logarithmic scale [30].

Analysis of 5'-US of *A. gambiae *immunity genes showed that the main motifs statistically overrepresented were those of 2, 3 and 4 nucleotides in length (Table 8). Similar to *A. gambiae*, *D. melanogaster *5'-US of immunity genes showed an enrichment of 2, 3 and 4 letter motifs (Table 8 and Tables 9, 10, 11, 12, 13, 14). Motifs of these length in 5'-US of immunity genes showed higher sig_occ _than those obtained in 5'-US of down-regulated and non-modified genes in both insects. For *A. gambiae *immunity genes, the highest score for 4 pb motifs was of 11.26 (ATAA), versus 2.59 (AAAA) and 2.46 (CGAC) in down-regulated and non-modified genes, respectively (Table 9). Motifs of 3 bp and 2 bp in length also present the highest sig_occ _in immunity induced *A. gambiae *genes: 6.11 for the AAA motif in immunity genes, versus 0.77 for the same motif in down-regulated genes, without 3 bp motif in non-modified genes (Table 11); 20.99 for AA motif in immunity genes, versus 0.72 and 1.73 for CG in down-regulated and non-modified genes, respectively (Table 13). Similar results for 2, 3 and 4 bp motifs were obtained in *D. melanogaster *5'-US (Tables 10, 12 and 14). These observations underline the high overrepresentation of certain motifs in 5'-US of these insect immunity genes. Interestingly, the motifs sequences with the highest scores in 5'-US of immunity genes were the same in both organisms: TA, AA, AT, AAA and ATAA (Table 8).

**Table 8:** Motif overrepresentation in 5' upstream sequences (2,500 bp) of immunity-induced genes.

Organism	Motif length (bp)	Motif	sig_occ_
***Anopheles gambiae***	2	**AA**	**20.99**
		**TA**	**14.39**
		**AT**	**13.25**
	3	**AAA**	**6.11**
		TCA	2.84
		AAC	2.17
	4	**ATAA**	**11.26**
		AATA	8.82
		TTAA	4.62
	5	AATAA	9.37
		ATAAA	5.1
		AATAC	3.7
	6	CAATAC	2.53
		AATAAT	2.19
		ATAATG	1.5
	7	AAAATAA	1.3
		ATTATTA	0.41
		AAATAAA	0.33

***Drosophila melanogaster***	2	**AA**	**37.43**
		**TA**	**15.64**
		**AT**	**14.8**
	3	**AAA**	**22.3**
		GAA	1.45
		AAG	0.95
	4	AAAA	16.61
		**ATAA**	**14.21**
		TAAA	9.59
	5	TAAAA	10.75
		AAAAT	8.29
		ATAAA	7.49
	6	TGATAA	5.97
		TAAAAA	5.64
		ATAAAA	5.36
	7	GAAAAAC	2.73
		TTAAAAA	2.39
		CTTATCA	2.03

First three motifs statistically overrepresented found with Oligo-Analysis for each motif length, in *A. gambiae *and *D. melanogaster *upregulated immunity genes. In bold are indicated motifs with high sig_occ _conserved in both insects.

**Table 9:** Motifs of 4 bp reported by Oligo-Analysis in 5'-US (2500 pb) of *A. gambiae *genes.

**Over-expressed immunity genes**		**Down-regulated genes**		**Non-modified genes**	
Motif	sig_occ_	Motif	sig_occ_	Motif	sig_occ_

ataa	11.26	aaaa	2.59	cgac	2.46
aata	8.82	aaat	1.38	ccgc	0.93
ttaa	4.62	caga	0.88	cggc	0.93
tcaa	4.02	cgcc	0.23	acgg	0.33
taaa	3.78			ccga	0.18
aatg	3.41				
atta	2.92				
aact	2.47				
atca	2.44				
atac	2.09				
aaat	1.77				
gtta	1.14				
tata	1.01				
attc	0.91				
acat	0.82				
aaaa	0.57				
aaag	0.37				
cata	0.36				
aaga	0.32				
gata	0.31				
atga	0.23				
ctta	0.18				
aaca	0.15				
attg	0.11				
atag	0.10				
taca	0.10				

**Table 10:** Motifs of 4 bp reported by Oligo-Analysis in 5'-US (2500 pb) of *D. melanogaster *genes.

**Immunity-induced genes**		**Down-regulated genes**		**Non-modified genes**	
Motif	sig_occ_	Motif	sig_occ_	Motif	sig_occ_

aaaa	16.61	ataa	2.20	caaa	0.65
ataa	14.21	ctaa	2.16	aaaa	0.31
taaa	9.59	ccag	1.03	aaac	0.18
aaat	9.15	ccca	1.00		
aata	7.66	atca	0.89		
tata	5.80	atgg	0.58		
gaaa	5.62	gata	0.48		
aatt	5.44	cgga	0.46		
atat	4.16	agat	0.38		
ttaa	3.28	taga	0.34		
atta	2.47	aatc	0.31		
aaag	2.34	gacc	0.18		
aatc	2.04	gaca	0.01		
aaac	1.70				
aaga	1.64				
tcaa	1.08				
agaa	0.98				
atca	0.66				
gtaa	0.63				
attc	0.52				
atga	0.26				
acaa	0.12				
caaa	0.02				

**Table 11:** Motifs of 3 bp reported by Oligo-Analysis in 5'-UR (2500 pb) of *A. gambiae *genes.

**Over-expressed immunity genes**		**Down-regulated genes**		**Non-modified genes**	
Motif	sig_occ_	Motif	sig_occ_	Motif	sig_occ_

aaa	6.11	aaa	0.77	none	
tca	2.84	gac	0.05		
aac	2.17				
caa	1.59				
taa	1.43				
ata	0.72				
act	0.58				
aat	0.44				
aca	0.36				

**Table 12:** Motifs of 3 bp reported by Oligo-Analysis in 5'-US (2500 pb) of *D. melanogaster *genes.

**Immunity-induced genes**		**Down-regulated genes**		**Non-modified genes**	
**Motif**	**sig**_occ_	**Motif**	**sig**_occ_	**Motif**	**sig**_occ_

aaa	22.3	cca	2.39	caa	1.7
gaa	1.45	acc	0.71	aaa	1.42
aag	0.95				
tca	0.89				

**Table 13:** Motifs of 2 bp reported by Oligo-Analysis in 5'-US (2500 pb) of *A. gambiae *genes.

**Over-expressed immunity genes**		**Down-regulated genes**		**Non-modified genes**	
Motif	sig_occ_	Motif	sig_occ_	Motif	sig_occ_

aa	20.99	cg	0.72	cg	1.73
ta	14.39			cc	1.46
at	13.25				

**Table 14:** Motifs of 2 bp reported by Oligo-Analysis in 5'-US (2500 pb) of *D. melanogaster *genes.

**Immunity-induced genes**		**Down-regulated genes**		**Non-modified genes**	
**Motif**	**sig**_occ_	**Motif**	**sig**_occ_	**Motif**	**sig**_occ_

aa	37.43	at	3.27	aa	2.97
ta	15.64	ta	2.25	at	0.11
at	14.8	cc	0.15		

The sig_occ _for the ATAA motif in 2500 pb 5'-US of *A. gambiae *immunity genes was 11.26, indicating that this four base-pairs motif is expected to occur in one of 10^11.26 ^groups with similar numbers of sequences of the same length of random sequences. In comparison, the ATAA motif was not present in non-modified and down-regulated genes of *A. gambiae *(Table 15 and Table 9). The ATAA motif in 5'-US *D. melanogaster *immunity genes had a sig_occ _of 14.21, versus 2.2 in down-regulated genes, and was absent in non-modified genes (Table 15 and Table 10). Similar results were obtained for the conserved motifs AAA, AA, AT and TA. These results indicate that statistical overrepresentation of these motifs is specific of immunity genes 5'-US (Table 15).

**Table 15:** Overrepresentation of AT rich motifs is specific of immunity-induced genes

**Organism**	**Group of genes**	**AA score**	**TA score**	**AT score**	**AAA score**	**ATAA score**
*Anopheles gambiae*	Immunity induced	20.99	14.39	13.25	6.11	11.26
	Down-regulated	---	---	---	0.77	---
	Non-modified	---	---	---	---	---
	Random genes	0.42	---	---	2.51	---
	Artificial	---	12.11	---	---	---

*Drosophila melanogaster*	Immunity induced	37.43	15.64	14.80	22.30	14.21
	Down-regulated	---	2.25	3.27	---	2.2
	Non-modified	2.97	---	0.11	1.42	---
	Random genes	1.9	---	---	0.7	---
	Artificial	---	---	---	---	---

The overrepresented motifs are specific of immunity-related gene promoters of *Anopheles gambiae *and *Drosophila melanogaster*.

**Figure 2:**
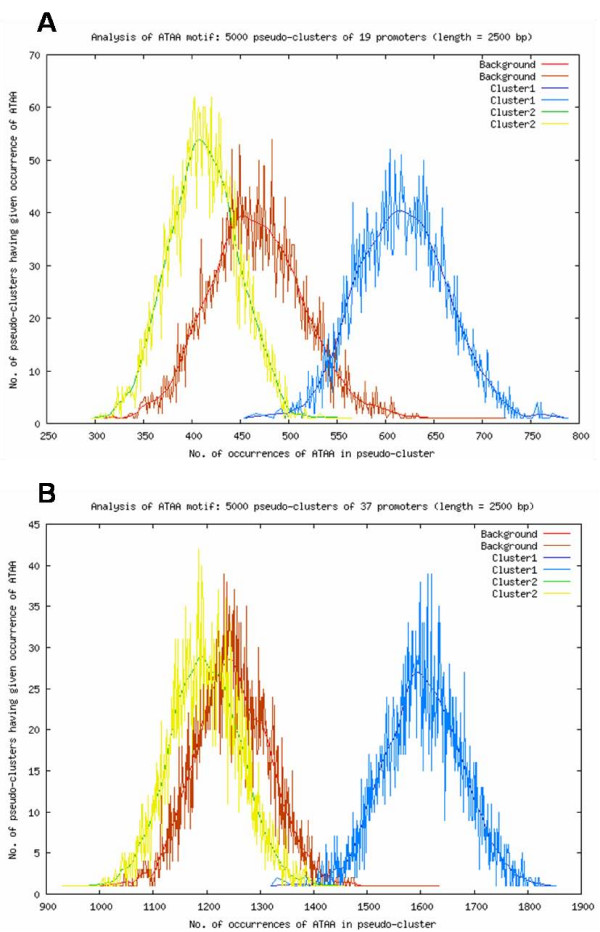
**Bootstrap to verify the ATAA enrichment in 5'-US regions of immunity-induced genes of *A. gambiae *and *D. melanogaster*.** In the vertical axis are the number of pseudoclusters having a given occurrence of the motif, in the horizontal axis are indicated the number of occurrences of the motif in pseudoclusters. The red curve represents ATAA enrichment in pseudoclusters generated from the complete genome of *A. gambiae *(**A**) or *D. melanogaster *(**B**), the blue curve represents ATAA enrichment in immunity-induced genes, the yellow and green curve represents ATAA enrichment in the non-modified genes group. Similar results were obtained for the TA, AA, AT and AAA motifs in both insects and versus the down-regulated genes.

In agreement to the Oligo-Analysis, bootstrapping analysis using POBO [31] confirmed that the average occurrence of the conserved motifs TA, AA, AT, AAA and ATAA, was significantly higher (p < 0.0001) in immunity promoters compared to the whole genome, the non-modified genes, down-regulated genes and the random sequence sets for both insects (Figure 2).

**Figure 3:**
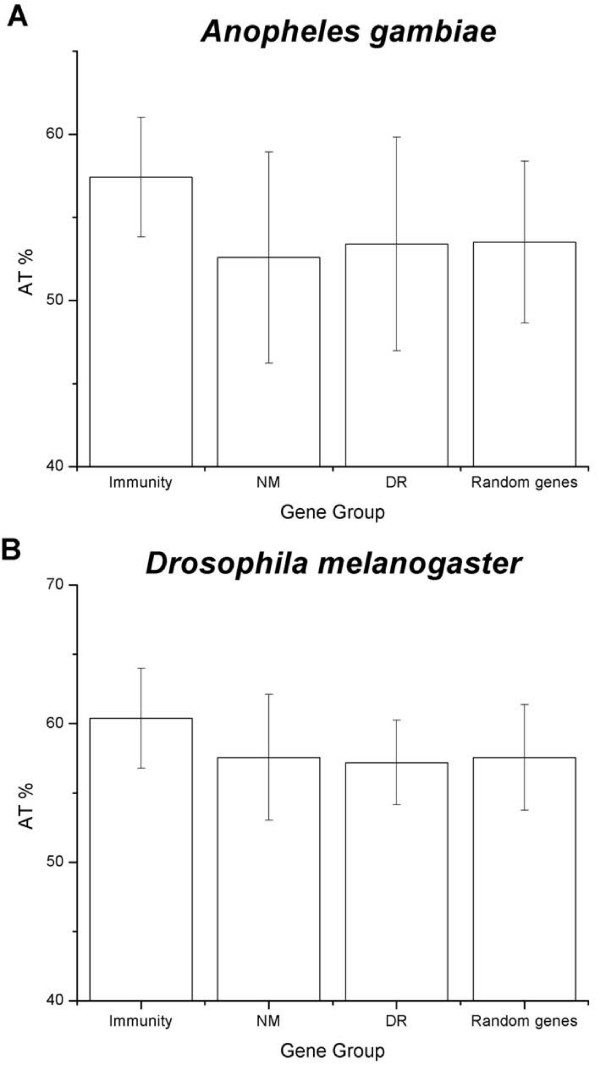
**Percentage of AT between the different groups of sequences of *A. gambiae *(A) and *D. melanogaster *(B).** The difference of AT% between groups is not statistically significant (p > 0.001), therefore, the enrichment of ATAA motif is not due to a bias in sequence composition. NM: non-modified genes, DR: down-regulated genes. Standard deviation is showed.

As a consequence of AT-rich motifs over-representation, a slight increase in AT% content was observed in 5'-US immunity genes, the average AT% for *D. melanogaster *immunity genes was of 60.4%, versus 57.6% and 57.2% for non-modified and down-regulated genes, respectively, these differences were not significant (p > 0.001), indicating that AT-rich motif over-representation was not due to a significant increase in AT % that could lead to a random AT-rich motifs enrichment. Similar results were obtained for *A. gambiae *(Figure 3).

### 5' upstream regions of immunity-related orthologous genes in the genus *Drosophila *and in *Aedes aegypti *are also enriched with AT-rich motifs

**Figure 4:**
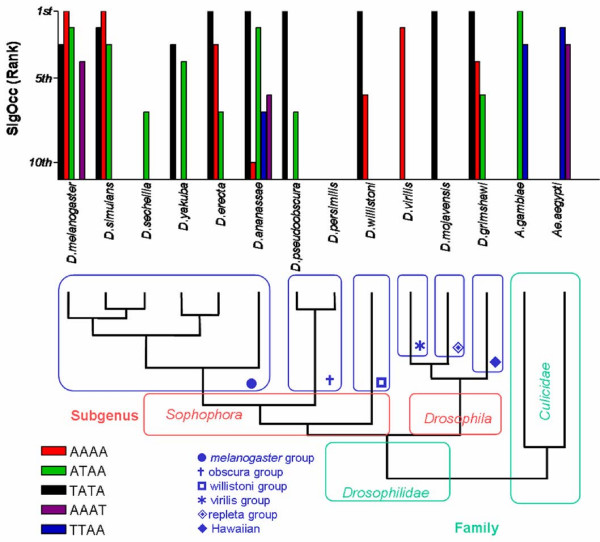
**Four bp AT-rich motif over-representation analysis in 5' US of dipteran immunity related induced genes and their corresponding phylogenetic relationships based on**[32]. Each AT-rich motif was plotted based on the rank obtained according to the corresponding Sig_Occ _for each genome.

In order to determine if other related dipteran species share the same motifs identified in *A. gambiae *and *D. melanogaster*, 5'-US of orthologous immunity genes of other *Drosophila *species and another *Culicidae *family member, *Aedes aegypti *were analyzed, regardless their transcriptional profile. Orthologous genes from those initially selected from *D. melanogaster *expression profiles were selected from the recently sequenced eleven *Drosophila *species [32,33]. Tables 16 and 17 show the list of orthologous genes present in each *Drosophila *species and *Ae. aegypti*. 5'-US from these genes were screened with Oligo-Analysis. Figure 4 shows the results obtained for the 4bp motifs TATA, AAAA, ATAA, AAAT and TTAA, which were statistically over-represented in the majority of 5'-US of immunity orthologous genes of 12 *Drosophila *species, *A. gambiae *and *Ae. aegypti*. We observed some phylogenetic correlations for some motifs. The most prevalent motif and with the highest sig_occ _scores was the TATA motif, which was within the best ranked for most *Drosophila *species (9/12), but absent in *Anopheles *and *Aedes*. The AAAA motif was also highly ranked among some *Drosophila *species but not in mosquitoes. The ATAA motif was highly ranked in *D. melanogaster*, *D. simulans *and *A. gambiae*, but absent in other *Drosophilas *and *Ae. aegypti*. Finally, motifs such as TTAA and AAAT were highly ranked in mosquitoes only. Intriguingly, *D. persimilis *did not show any enrichment of AT-rich motifs at all, regardless its close genetic distance to species displaying clear AT-rich motif enrichment. It should be noted that Sig_occ _values for the non-melanogaster sequences and *Ae. aegypti *were considerably lower than those observed in *A. gambiae *and *D. melanogaster *(Table 18. See additional file 1).

**Table 16:** List of Drosophilid orthologous immunity genes used for over-representation analysis of AT-rich tetrads in 5' upstream regions (2000 bp)

**Subgenus *Sophophora***									**Subgenus *Drosophila***		
***melanogaster group***						***obscura group***		***willistoni group***	***virilis group***	***repleta group***	***Hawaiian***

***melanogaster***	***simulans***	***sechellia***	***yakuba***	***erecta***	***ananassae***	***pseudoobscura***	***persimilis***	***willistoni***	***virilis***	***mojavensis***	***grimshawi***

FBgn0012042	*Dsim\AttA*	*Dsec\AttA*	*Dyak\AttA*			*Dpse\GA10109*					
FBgn0030310	*Dsim\PGRP-SA*	Dsec\GM13088	*Dyak\PGRP-SA*			*Dpse\GA11152*	Dper\GL26887	Dwil\GK25449	Dvir\GJ15950	Dmoj\GI16473	
FBgn0000276	*Dsim\CecA1*	*Dsec\CecA1*				*Dpse\CecI*					
FBgn0000279	*Dsim\CecC*	*Dsec\CecC*	*Dyak\CecC*	*Dere\CecC*		*Dpse\CecV*					
FBgn0041581	*Dsim\AttB*					*Dpse\GA14910*					
FBgn0000277			*Dyak\CecA2*			*Dpse\CecIII*			*Dvir\Cec2A*		
FBgn0025583						*Dpse\GA14796*					
FBgn0011274	*Dsim\Dif*	Dsec\GM17131	*Dyak\Dif*	Dere\GG21748	Dana\GF15105	*Dpse\GA19867*	Dper\GL18755	Dwil\GK24830	Dvir\GJ16233	Dmoj\GI16552	Dgri\GH10078
FBgn0004240	Dsim\GD11417	Dsec\GM21923	*Dyak\Dpt*	Dere\GG21934	Dana\GF11124	*Dpse\GA11797*	Dper\GL11494	Dwil\GK20931	Dvir\GJ19915	Dmoj\GI20150	Dgri\GH22131
FBgn0000462	*Dsim\dl*	Dsec\GM17128	*Dyak\dl*	Dere\GG21746	Dana\GF15103	*Dpse\GA19765*	Dper\GL18753	Dwil\GK10525	Dvir\GJ16232	Dmoj\GI16541	Dgri\GH10077
FBgn0035434	*Dsim\dro5*	Dsec\GM14562	*Dyak\dro5*	*Dere\dro5*							
FBgn0035976	Dsim\GD14166	Dsec\GM25129	Dyak\GE20818	Dere\GG15356	Dana\GF10680	*Dpse\GA29295*	Dper\GL18427	Dwil\GK23800	Dvir\GJ13383	Dmoj\GI12108	Dgri\GH15170
FBgn0035977	Dsim\GD14168	Dsec\GM25131	Dyak\GE20821	Dere\GG15359	Dana\GF10681	*Dpse\GA18183*	Dper\GL18430	Dwil\GK23822	Dvir\GJ13386	Dmoj\GI12111	Dgri\GH15173
FBgn0034328		Dsec\GM19911	Dyak\GE13917	Dere\GG20976	Dana\GF12769	Dpse\GA24273	Dper\GL16707	Dwil\GK23237	Dvir\GJ22454	Dmoj\GI19391	Dgri\GH20222
FBgn0003717	*Dsim\Tl*	Dsec\GM10345	*Dyak\Tl*	Dere\GG11504	Dana\GF16456	*Dpse\Tl*		Dwil\GK13544	*Dvir\Tl*	Dmoj\GI22147	Dgri\GH14238
FBgn0014018	*Dsim\Rel*	Dsec\Rel	*Dyak\Rel*	Dere\GG17396	Dana\GF18430	*Dpse\GA11317*	Dper\GL12490	Dwil\GK14061	Dvir\GJ23481	Dmoj\GI10193	Dgri\GH18237
FBgn0010381	*Dsim\Drs*	Dsec\GM14569	*Dyak\GE21361*	*Dere\GG15135*							
FBgn0043575	*Dsim\PGRP-SC2*	Dsec\GM21061	*Dyak\GE19223*	*Dere\GG23381*		*Dpse\GA13217*		Dwil\GK21737	Dvir\GJ21836	Dmoj\GI18809	
FBgn0034329						*Dpse\GA14798*					
FBgn0034407	Dsim\GD11419	Dsec\GM21924	Dyak\DptB	Dere\GG21935	Dana\GF11125	*Dpse\GA10563*	Dper\GL11495	Dwil\GK20932	Dvir\GJ19917	Dmoj\GI19362	Dgri\GH22132
FBgn0010388	*Dsim\Dro*	Dsec\GM21566	Dyak\GE13605	Dere\GG20474	Dana\GF11338	*Dpse\GA10577*	Dper\GL11457	Dwil\GK19342			
FBgn0028990	Dsim\GD22555	Dsec\GM14001	Dyak\GE18418	Dere\GG23599	Dana\GF14458	*Dpse\GA10926*	Dper\GL25590	Dwil\GK18897	Dvir\GJ21412	Dmoj\GI24139	Dgri\GH13574
FBgn0010385	*Dsim\Def*	Dsec\GM20557	Dyak\GE21830	Dere\GG25236	Dana\GF12914	*Dpse\GA12570*	Dper\GL10559	Dwil\GK21361	Dvir\GJ22479	Dmoj\GI19416	Dgri\GH21581
FBgn0037906	Dsim\GD15111	Dsec\GM26096	Dyak\GE24618	Dere\GG17219	Dana\GF16643	*Dpse\GA13189*	Dper\GL12268	Dwil\GK11791	Dvir\GJ10054	Dmoj\GI22456	Dgri\GH15357
FBgn0033835						*Dpse\GA14871*					
FBgn0002930	*Dsim\nec*	Dsec\GM20812	*Dyak\nec*	Dere\GG10764	Dana\GF12243	*Dpse\GA14995*	Dper\GL10947	Dwil\GK21526	Dvir\GJ14938	*Dmoj\GI20049*	Dgri\GH20376
FBgn0000278	*Dsim\CecB*	Dsec\CecB	Dyak\GE10866	Dere\GG11950	Dana\GF16190						
FBgn0041579	*Dsim\AttC*	Dsec\GM21465	Dyak\GE12538	Dere\GG20378	Dana\GF13689			Dwil\GK17822	Dvir\GJ21173		Dgri\GH21628
FBgn0000250	*Dsim\cact*	Dsec\GM17910	*Dyak\cact*	Dere\GG24172	Dana\GF15324	*Dpse\GA19176*	Dper\GL14084	Dwil\GK23715	Dvir\GJ17162	Dmoj\GI14683	Dgri\GH11429
FBgn0010441	*Dsim\pll*	Dsec\GM10117	*Dyak\pll*	Dere\GG12125	Dana\GF18719	*Dpse\GA19272*	Dper\GL23050	Dwil\GK22593	Dvir\GJ10484	Dmoj\GI22298	Dgri\GH16651
FBgn0035806	*Dsim\PGRP-SD*		*Dyak\PGRP-SD*	Dere\GG14444	Dana\GF10655	*Dpse\GA20392*	Dper\GL26612	Dwil\GK23884	Dvir\GJ11481	Dmoj\GI12249	Dgri\GH14703
FBgn0038530	Dsim\GD19176	Dsec\GM15251	*Dyak\AttD*	Dere\GG22395	Dana\GF17819	*Dpse\GA20491*	Dper\GL21518	Dwil\GK12085	Dvir\GJ22662	Dmoj\GI24598	Dgri\GH18099
FBgn0014865	*Dsim\Mtk*	Dsec\GM21609	Dyak\GE11702	Dere\GG20517		*Dpse\GA20868*		Dwil\GK19217			
FBgn0022073	*Dsim\Thor*	Dsec\GM11143	*Dyak\Thor*	Dere\GG24948	Dana\GF15239	*Dpse\GA21364*	Dper\GL18656	Dwil\GK18436	Dvir\GJ17238	Dmoj\GI15007	Dgri\GH10214
FBgn0043578	*Dsim\PGRP-SB1*	Dsec\GM24370	Dyak\GE22193	Dere\GG15854	Dana\GF10506	*Dpse\GA21961*	Dper\GL13224	Dwil\GK17343	Dvir\GJ12160	Dmoj\GI11935	Dgri\GH15225

Underlined: Genome-wide drosophilid orthologs.

*Italic*: Curated drosophilid ortholog

**Table 17:** *Aedes aegypti *orthologous immunity genes used for over-representation analysis of AT-rich motifs in 5'-US (2500 bp)

**Ensembl Gene ID**	**Description**
AAEL000621	antibacterial peptide, putative
AAEL000709	developmental protein cactus
AAEL001794	macroglobulin/complement
AAEL001802	macroglobulin/complement
AAEL002972	brain chitinase and chia
AAEL003889	gram-negative bacteria binding protein
AAEL004522	Orthologous of putative infection responsive short peptide
AAEL005787	serine protease, putative
AAEL007765	serine protease inhibitor 4, serpin-4
AAEL008646	fibrinogen and fibronectin
AAEL009423	cd36 antigen
AAEL009520	Leucine rich domain
AAEL010171	peptidoglycan recognition protein sb2
AAEL010737	aromatic amino acid decarboxylase
AAEL014238	aromatic amino acid decarboxylase

### AT-rich tetrads are associated with high nucleosomal potential

Once observed that AT-rich motif enrichment was a general feature of 5'-US of immunity genes in several dipteran species, we evaluated the association of some of these motifs with predicted nucleosomal sites. Experimentally stable nucleosomes in mouse have AT-rich motifs, including the AA [20], TA [14,20], TATA and ATAA motifs [18]. The ATAA motif include the three 2 bp motifs statistically over-represented in immunity genes (AA, TA and AT) of *A. gambiae *and *D. melanogaster*. Taking into account the highly conserved nucleosomal structure and given that the ATAA motif was enriched in both *D. melanogaster *and *A. gambiae*, as well as in other *Drosophila *species, we hypothesized that the ATAA motif could also participate in nucleosome formation in dipteran immune response genes. Some algorithms have been developed to predict the chromatin structure from sequence [20,22,34]. The RECON algorithm uses experimentally determined nucleosomal sequences coupled to Monte Carlo methods and discriminant analysis of dinucleotide frequencies [22]. It searches for a partition of non-overlapping regions in the nucleosomal sequences that provides the maximal value of the Mahalanobis distance that discriminates between nucleosomal and non-nucleosomal sequences. In this way, RECON determines the probability that a sequence forms nucleosomes and assign a nucleosomal potential value to each nucleotide according to the context of the sequence in which the nucleotide is immersed. Positive values of nucleosomal potential correspond to reliable predictions of nucleosome formation sites with a confidence level of p < 0.05 (α = 0.05), nucleosomal potential of +1 corresponds to the best predictions.

**Figure 5:**
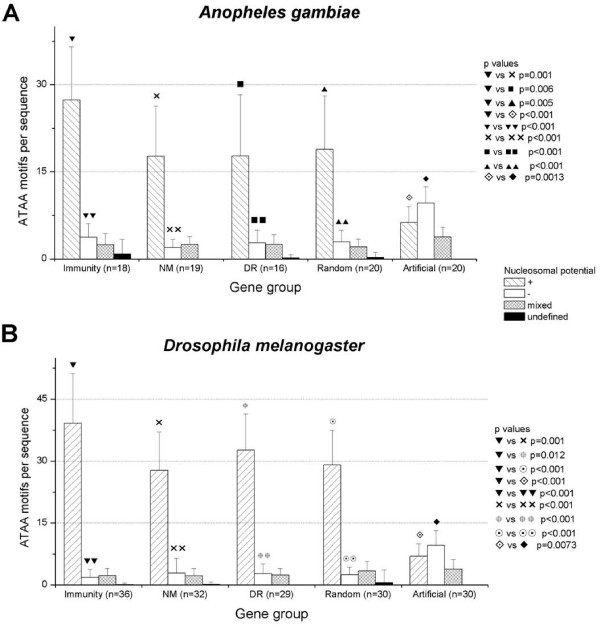
**ATAA motifs are associated preferentially with regions of positive nucleosomal potential in *A. gambiae *(A) and *D. melanogaster *(B).** ATAA motifs are present in all the groups of sequences analyzed, however 5'-US of immunity genes have more ATAA per sequence. ATAA distribution differ between biological and non-biological (artificial) sequences, which have less ATAA motifs and are associated primarily with negative values of nucleosomal potential. Standard deviation is showed. (NM:non-modified, DR:down-regulated, Random: random selected genes, Artificial: computer-derived non-biological sequences).

Using RECON, ATAA motifs were preferentially associated to positive values of nucleosomal potential in all the biological groups, both in *A. gambiae *and *D. melanogaster *(Figure 5), supporting a possible role for this motif in nucleosome formation. From Figure 5 is evident that ATAA is associated with positive nucleosomal potential values independently of the group of biological 5'-US analyzed. As expected, immunity, down-regulated, non-modified and random selected 5'-US of *A. gambiae *and *D. melanogaster*, all have ATAA motifs, however, 5'-US of immunity genes have a significant increased number of ATAA motifs. The frequency of ATAA associated with positive nucleosomal potential in immunity 5'-US is higher than non-modified (p = 0.001 both in *A. gambiae *and *D. melanogaster*), down-regulated (p = 0.006 in *A. gambiae *and p = 0.012 in *D. melanogaster*), random (p = 0.005 in *A. gambiae *and p < 0.001 in *D. melanogaster*) and non-biological (artificial) sequences (p < 0.001 in *A. gambiae *and *D. melanogaster*). Analyzing ATAA distribution per group of genes, more than 70% and 80% of all ATAA motifs in *A. gambiae *and *D. melanogaster*, respectively, were located within regions of positive nucleosomal potential values (p < 0.001), indicating a possible role of this motif in nucleosome formation.

Additional information derived from Figure 5 is that the combination of RECON with Oligo-Analysis results allows detection of a property inherent to biological sequences. The ATAA distribution with respect to nucleosomal potential values was utterly different between biological and non-biological (artificial) sequences for both insects. On the one hand, biological sequences had more ATAA than non-biological sequences (1065 ATAA motifs in 30 random biological sequences versus 613 in 30 random non-biological sequences). The majority of the biological ATAA motifs were associated to positive nucleosomal potential (872/1065 or 81.9% motifs in 30 random biological sequences versus 208/613 or 33.9% in 30 random non-biological sequences). Additionally, the non-biological sequences presented an inverse distribution of ATAA motifs, with more ATAA motifs associated to negative values of nucleosomal potential (289/613 or 47.2% ATAA negatives versus 208/613 or 33.9% ATAA positives in 30 random non-biological sequences).

**Figure 6:**
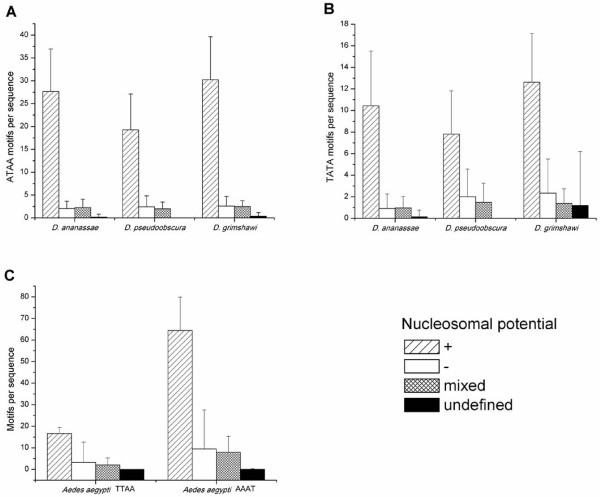
**AT-rich motifs in the family *Drosophilidae *and *Culicidae *are associated preferentially with regions of positive nucleosomal potential calculated by the RECON algorithm**[22]. ATAA (A) and TATA (B) motifs are associated with positive nucleosomal potential in *D. ananassae*, *D. pesudoobscura *and *D. grimshawi *(p < 0.001), which belong to different groups. Over-represented TTAA and AAAT motifs in *Ae. aegypti *are also associated preferentially with regions of positive nucleosomal potential (C, p < 0.001). Standard deviation is showed.

We also evaluated the association of ATAA and TATA motifs in *D. ananassae*, representing the non-melanogaster subgroup; *D. pseudoobscura *of the *obscura *group and *D. grimshawi*, the most phylogenetically distant species belonging to the Hawaiian *Drosophila*, as well as the TTAA and AAAT motif in *Ae. aegypti *(non-drosophilid dipteran), with nucleosomal potential calculated by the RECON algorithm. As shown in Figure 6, both motifs in the drosophilid species analyzed are clearly associated to positive nucleosomal potential values (p < 0.001; Figure 6A–B). Similarly, both motifs analyzed in *Ae. aegypti *were associated to high nucleosomal potential values and the difference between AT-rich motifs with positive nucleosomal potential and AT-rich motifs with other values was statistically significant (p < 0.001) (Figure 6C).

### ATAA motifs correlate with high Nucleosomal Occupancy *p *values (pNO)

Segal and col. [20] recently reported an algorithm to predict nucleosome positions that takes into account sequence composition and thermodynamic properties. Using a collection of nucleosome bound DNA sequences from yeast, chicken or human, they constructed probabilistic models that represent the DNA sequence preferences for nucleosome formation and assign a *p *value to each nucleotide of the analyzed sequence; this value indicates the probability that the position is occupied by a nucleosome (p of Nucleosomal Occupancy, pNO).

**Figure 7:**
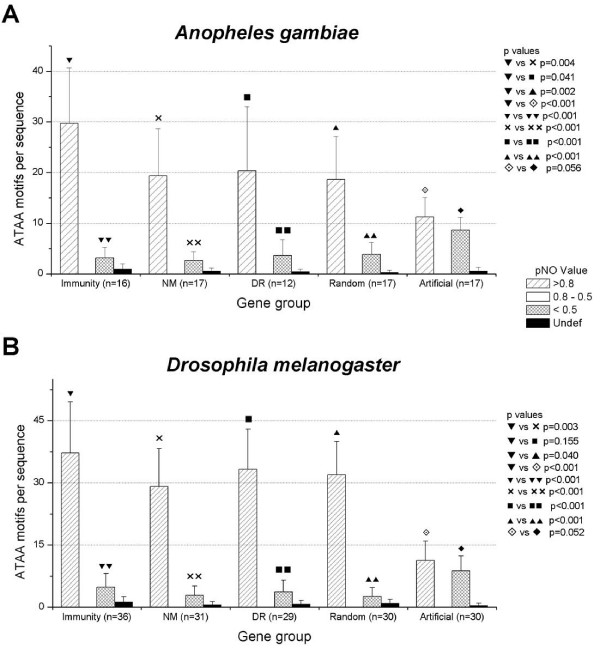
**ATAA motifs are associated with high p values of nucleosomal occupancy (pNO), in *A. gambiae *(A) and *D. melanogaster *(B), evaluated with the software "Nucleosomes Positioning", reported by Segal and col.**[20]**, when the yeast model is applied.** None ATAA motif was associated with pNO values between 0.8 and 0.5. Non-biological sequences (artificial) show an ATAA distribution different to biological sequences. Note the similarity between this figure and figure 5. Standard deviation is showed.

Applying the three models to all the groups of sequences analyzed from *A. gambiae *and *D. melanogaster*, we found that the ATAA motifs were associated with high pNO values when the sequences were analyzed using the yeast model (Figure 7). Distribution of ATAA was very similar to that obtained using RECON, showing a coincidence between two independent methods to predict the association of ATAA with nucleosomal positions. All the biological groups of analyzed sequences presented ATAA motifs associated largely with pNO > 0.8, however, immunity genes had a significant increased number of ATAA associated with pNO > 0.8 values in relation to other biological sequences (p < 0.05, except between immunity and down-regulated genes of *D. melanogaster*, where p = 0.155).

More than 85% of all ATAA motifs found in 5'-US of *A. gambiae *and *D. melanogaster *were associated with pNO > 0.8 (p < 0.001). For *A. gambiae *immunity genes, 88% (476/541) of ATAA motifs were associated with pNO > 0.8, a similar distribution was obtained for the other *A. gambiae *gene groups (Figure 7A). The difference between ATAA associated with pNO > 0.8 and ATAA associated with pNO < 0.5 or undefined values was statistically significant (p < 0.001), showing a clear correlation between ATAA and high values of pNO. In a similar way, 85.9% (1340/1560) of ATAA motifs in 5'-US *D. melanogaster *immunity genes had pNO > 0.8, with a significant difference with regard to ATAA with pNO < 0.5 or undefined values (p < 0.001), the distribution of ATAA in the other *D. melanogaster *groups of genes also was statistically significant (p < 0.001) (Figure 7B).

The combination of Oligo-Analysis and pNO results also revealed a difference between biological and non-biological sequences. Thirty randomly selected biological sequences of *D. melanogaster *had 90% (959/1065) of ATAA motifs associated with pNO > 0.8 versus 55.1% (338/613) of ATAA motifs in 30 non-biological sequences, showing again a non-random distribution of biological ATAA motif and tagging it as part of a potential nucleosomal code (Figure 7B).

Surprisingly no ATAA motifs were found with pNO values between 0.8 and 0.5 (values that define the "medium p value" range, see methods) in any of the other gene groups analyzed in *A. gambiae *and *D. melanogaster *(Figure 7).

**Figure 8:**
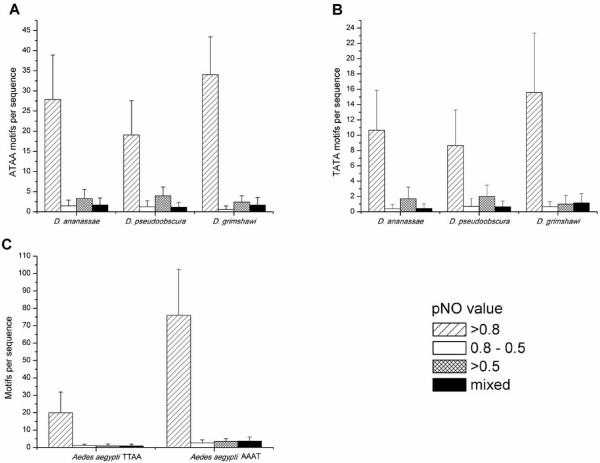
**AT-rich motifs in *Drosophilidae and Culicidae *families are associated with high p values of nucleosomal occupancy (pNO), calculated according to Segal, *****et al ***[20]**using the yeast model for *D. ananassae*, *D. pseudoobscura *and *D. grimshawi *(A and B) and *Ae. aegypti *(C).** pNO is plotted in relation to its association with ATAA (A) and TTAA (B) or TTAA and AAAT (C) motif frequency. In every species, the AT-rich motif analyzed was associated to high pNO. Standard deviation is showed.

We evaluated the association to probability of nucleosomal occupancy (pNO) [20] of ATAA and TATA motifs in *D. ananassae*, *D. pseudoobscura *and *D. grimshawi*; and the TTAA and AAAT motif in *Ae. aegypti*. As shown in Figure 8, both motifs in the drosophilid species analyzed are clearly associated to pNO > 0.8 values (p < 0.001, Figures 8A–B). Similarly, both motifs analyzed in *Ae. aegypti *were associated to pNO > 0.8 values (p < 0.001, Figure 8C).

Taken together, we found a consistent tendency, demonstrated by two independent methods, showing that the AT-rich motif enrichment within a specific sequence context might favour nucleosome formation in immune genes of a wide variety of dipteran species.

### *A. gambiae *and *D. melanogaster *5'-US of immunity genes have NFκB response elements located in the first 500 pb of their 5'-US

**Figure 9:**
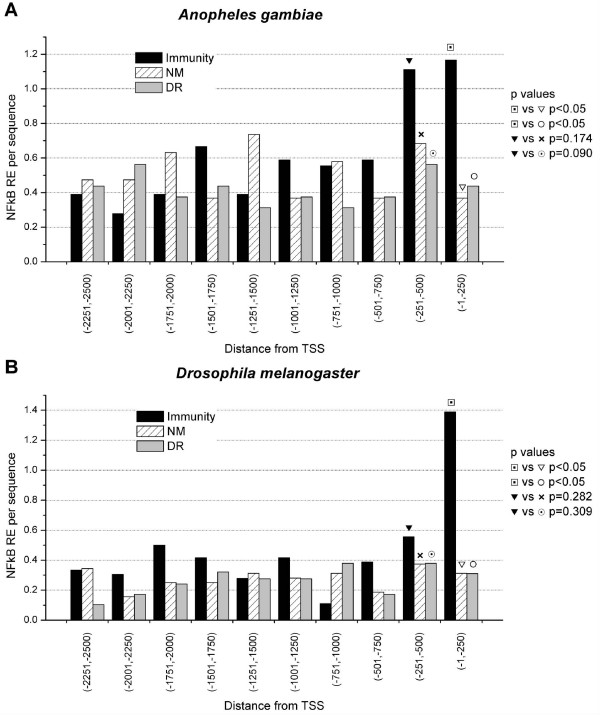
**5'-US of immunity genes have NFκB response elements located mainly in the first 500 bp upstream the transcription initiation site.** Non-modified (NM) and down-regulated (DR) genes do not present this enrichment in NFκB motifs.

**Figure 10:**
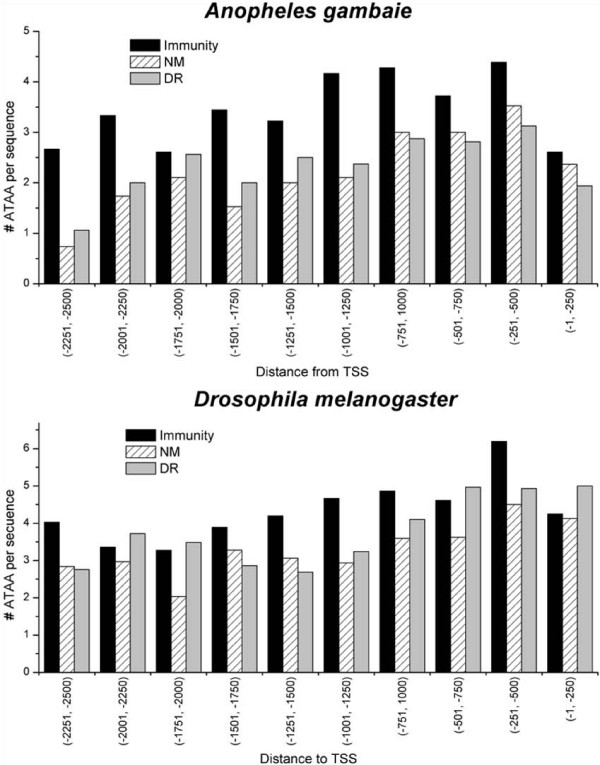
ATAA motifs are located predominantly in the -251 to -500 region of immunity genes in *A. gambiae *and *D. melanogaster*, showing a coincidence with regions where NFκB RE are more abundant.

**Figure 11:**
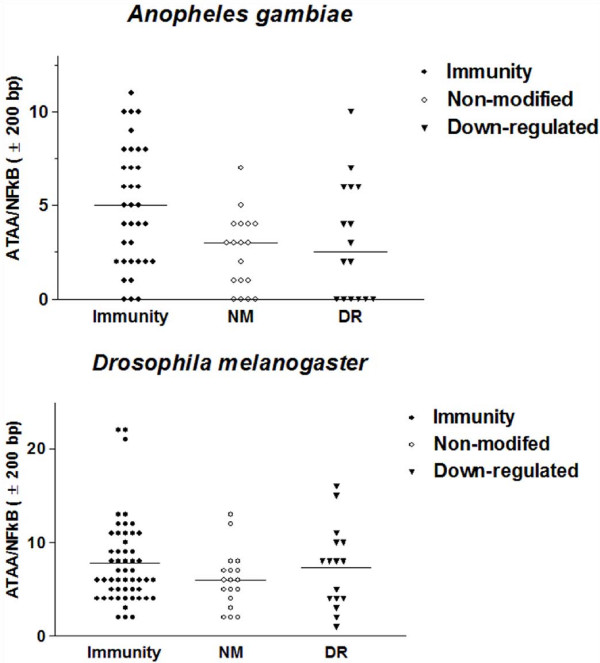
There are more ATAA motifs around NFκB REs of immunity genes than around NFκB RE's of non-modified (NM) and down-regulated (DR) genes.

NFκB transcription factors in both insects and vertebrates are involved in immune gene expression regulation [23,25]. Using MEME [35], NFκB REs were identified in the 5'-US of immunity genes, but not in 5'-US of down-regulated, non-modified and random genes. Moreover, the NFκB REs were enriched within the first 500 bp of the 5'-US of immunity genes, both in *A. gambiae *and *D. melanogaster *(Figure 9). In order to investigate if there is a functional and physical association between ATAA motifs and NFκB motifs, the distribution of ATAA motifs was analysed with respect to the transcription initiation site (TIS). Although ATAA motifs were distributed along the whole sequence in all gene groups, the highest frequencies were found to be located within the -251 to -500 interval in immunity genes in both insects (Figure 10). We further quantified ATAA frequency within ± 200 bp from the NFκB site of immunity, NM and DR genes in both insects. In the case of *A. gambiae*, the frequency of ATAA motifs around NFκB REs is significantly higher in immunity genes compared to both non-modified (p < 0.01) and down-regulated genes (p = 0.048). However, in the case of *D. melanogaster *we did not find significant differences in the ATAA frequency in relation to NFκB RE among the gene groups (p > 0.1), although the tendency was equal to that of *A. gambiae *(Figure 11). The association between NFκB REs to AT-rich motifs and possibly nucleosomes of immunity genes of *A. gambiae *and *D. melanogaster *may function as a specific link between the chromatin structure and the remodelling machinery needed for the expression of immune response genes.

## Discussion

In this work, we have documented that AT-rich motifs are over-represented in 5' upstream regions of immunity genes of mosquitoes and drosophilids. We documented also that the position of the AT-rich motifs is associated to nucleosomes as predicted by two different algorithms for nucleosome positioning, pointing out to a possible role of this motif in the transcriptional regulation of these functionally related genes through modification of chromatin structure involving nucleosome positioning.

Previous reports have found that sequences that form extremely stable nucleosomes are enriched with AT motifs referred as TATA boxes, which in many cases included the ATAA motif [18]. When we correlated the positions of this motif with the output of two different algorithms that predict nucleosome positions [20,22], we found that this motif correlates almost exclusively with positions with a high probability to form nucleosomes, suggesting that the ATAA motif enrichment is a DNA sequence pattern associated to nucleosome formation in these functionally related immunity genes. The conservation of enrichment of AT-rich motifs in 5'-US of immunity-related genes of *Drosophilidae *and *Culicidae*, which diverged 250 million years ago [36], suggest that this common feature may be the result of evolutionary constrained epigenetic mechanism of transcriptional regulation in immune-responsive genes in dipterans. More studies are required to define if this could be part of a more general mechanism of regulation in metazoans. The case of *D. persimilis *represents a caveat for our attempt generalize the implications of our findings, however, we cannot exclude that the current status of the annotation of such genomes may affect the results.

There are two conflicting views about nucleosome formation: one establishes that nucleosomes can potentially be formed anywhere in the genome regardless the sequence and therefore, it is not possible to predict sites for nucleosome formation [4]. The other proposes that nucleosomes are associated to certain DNA sequences or sequence patterns that have an effect on the bending properties of DNA during nucleosome formation [14,18]. This point of view has been gaining support in recent years due to the documentation of a great variability in the bending potential of DNA sequences [14,15,37] and therefore their capacity to form nucleosomes [18,38,39].

Two of the three two-letter motifs statistically over-represented in immunity promoters of *A. gambiae *and *D. melanogaster*, TA and AA, have been previously associated to nucleosome formation in human, yeast, chicken and mouse [14,15,20,21]. Additionally, the ATAA motif, which is associated to nucleosome positions, and includes the three motifs containing two letters with the highest scores in both organisms (AA, TA and AT), have been found in sequences that form stable nucleosomes [18]. Thus, AT-rich motifs in 5'-US regions of mosquitoes and drosophilid immunity genes could participate in the transcriptional regulation of genes induced by immune challenges in a different way to the typical response elements. In contrast to response elements, which can be functional single or in pairs in a promoter region, the AT-rich motifs are statistically enriched, with several copies distributed in a diffuse pattern through the promoter regions, suggesting its involvement in nucleosome formation. This diffuse sequence pattern of AT-rich motifs, different to discreet patterns displayed by response elements, represents a new insight on the role of DNA sequence context in transcriptional regulation.

Several reports have documented that genes with similar functions share similar nucleosomal occupancy patterns. Levitsky and col. [34], using the RECON algorithm to analyze distinct functional types of human promoters, found that tissue-specific gene promoters present higher nucleosomal potential than genes commonly expressed in many tissues (housekeeping genes). Segal and col. [20], using the Nucleosome Position Prediction algorithm to analyze different kinds of genomic sequences and gene sets biologically related, found that nucleosome occupancy varies depending of the analyzed genomic location type, and that groups of genes functionally related can be classified on the basis of their profiles of nucleosome occupancy in the open reading frames and intergenic regions. Recently, Lee and col. [40], using Hidden Markov Models to analyze experimentally obtained nucleosomes, also found a correlation between function and nucleosome occupancy. Each of these reports used a different method to analyze data sequences, and all found that nucleosomal sequences follow a distinctive pattern associated to the functionality of the genes.

In relation to RECON [22] and Nucleosome Positioning Prediction [20], it is important to note that none of these programs search for *a priori *defined motifs, the input for both programs are biological nucleosomal sequences from which information is extracted.

It has been shown that gene expression co-regulation is highly conserved in eukaryotes, for example, *Saccharomyces cerevisiae *and *Caenorhabditis elegans*, which diverged 1500 million years ago, still share a group of co-regulated genes [41], so it is plausible that *Drosophilidae *and *Culicidae *which diverged only 250 million years ago also share groups of functionally related co-regulated genes. The enrichment of AT-rich motifs in groups of co-regulated genes involved in immune response could provide the basis for developing new tools for the identification of different functional gene modules based on the compositional context of non-coding regulatory DNA. However, the high sig_occ _observed in manually curated datasets compared to the low sig_occ _observed in automatically annotated datasets highlights the importance of accurate TIS for regulatory region analysis.

Insect immunity relies on innate defense mechanisms to combat pathogens. In *D. melanogaster*, the Imd and Toll pathways lead to the activation of Rel/NFκB transcription factors that control a substantial proportion of the transcriptionally modified genes in response to pathogen infection [42]. Many components of these pathways are conserved in *A. gambiae *and *Ae. aegypti *[43] and are also remarkably conserved in innate immunity signaling pathways in mammals (TLR and TNF-R signaling pathways, respectively) [33,44-47]. The set of induced genes in both insects described here belong to the same functional group and many of them have NFκB response elements within 500 pb upstream from the predicted transcription start site, the same location where functionally important NFκB REs have been found in these and other insects [48-52]. Our findings indicate that besides being regulated by NFκB, the enrichment with the ATAA motif constitutes a particular pattern of chromatin structure involved in transcriptional regulation of these genes.

Interestingly, NFκB transcription factors bind to their response elements even if they are packaged in a nucleosome [53]. Once bound to their response elements, NFκB transcription factors can recruit chromatin remodeling complexes to expose other response elements and allow the formation of the initiation complex [54]. In the vertebrate immune system, chromatin structure is critical to establish Th1-Th2 differentiation through the action of specific transcription factor as GATA-3 and T-bet [55], and several cytokines posses nucleosomes located in their promoters which need to be removed to allow gene expression [56-59]. Thus, epigenetic phenomena such as histone modification (altered nucleosome conformation) [60] or remodeling of chromatin (change of nucleosome position) [61] are commonly a required step to achieve gene expression in response to external stimuli.

Based on the obtained results and previously reported information, we propose a model in which a subgroup of insect immunity genes remains silent in absence of an immune challenge due to nucleosome formation in their 5'-US regions. The presence of these nucleosomes occludes the access of transcription factors to REs involved in gene expression. After an immune challenge, the Toll and/or Imd pathways are activated which in turn lead to activation of Rel/NFκB transcription factors, which are translocated to the nucleus and bind to their NFκB REs and recruit chromatin modifying/remodeling factors that release DNA from nucleosomes allowing its interaction with the transcriptional machinery.

Functionally related genes could harbor in their regulatory region a regulatory code represented by the combination of REs plus, in some cases, particular short motifs associated to chromatin structure. This regulatory code functions like a lock, genes that need to be co-expressed will share the same lock, represented by REs organized in a similar way, or by specific REs associated to motifs that confer a distinctive chromatin structure. Cells are continuously sensing its environment and responding to adapt. The regulatory state of the cell, defined by the presence and state of activity of transcription factors [3], also changes continuously; this regulatory state represents the "key" needed to open the proposed lock. The active transcription factors present in a given time in the cell, determines the form of the "key" for the lock, and therefore, the class of promoters that will be open or closed. In the case of the immune genes studied here, we have identified evidence that is compatible with a potential regulatory unit involving chromatin structure (associated with ATAA), Rel/NFκB transcription factors and NFκB response elements. Other regulatory codes could exist involving anyone of these components, in addition to others.

The role of chromatin structure in gene expression regulation during immune response of insects remains poorly explored. This work provides a first insight into this complex regulatory mechanism potentially shared by immune genes of *drosophilidae *and *culicidae*.

## Conclusion

Immunity genes of *A. gambiae, Ae. aegypti*, *D. melanogaster *and many other Drosophilid species share a common enrichment of AT-rich motifs in their 5'-US regions. AT-rich motifs are frequently associated to bioinformatic nucleosome positioning predictions, suggesting their participation in a particular nucleosome organization involved in transcriptional regulation of an immunity co-regulated module. Many of these regulatory regions also have NFκB response elements within the first 500 bp 5' from the transcription start site. These two features suggest that the mechanism of transcriptional regulation of immune response genes in dipterans are conserved and might occur through modifications in chromatin structure of promoter regions mediated by NFκB-dependent recruitment of remodeling factors. Our findings suggest that AT-rich motif enrichment in regulatory regions in this group of co-regulated genes could represent an evolutionary constrained signature in dipterans and perhaps other species, despite their evolutionary distance.

## Methods

### Gene selection criteria

Microarray data for *A. gambiae *immune response was kindly provided by the author [26]. Microarray data from *D. melanogaster *immune response [27] was downloaded from [62]. Analysis of expression profiles was conducted using the TMEV version 3.1 module of TM4 microarray software suite [63].

Three gene clusters were selected for each species by hierarchical clustering [64] from the microarray databases. For *A. gambiae*, gene selection criteria were as follows: **Induced immunity-related genes**: genes associated with immunity based on the protein structural features, and with expression values in log_2_(f2/f1) > 0 in at least 7/10 immunological challenges and 3/6 points for each challenge. **Non-modified genes**: genes with an expression mean of log_2_(f2/f1) ± 0.10 and a standard deviation of ± 0.15 in 9/10 immunological challenges. **Down-regulated genes**: genes with an expression level of log_2_(f2/f1) ≤ -0.5855 (a repression level of at least 1.5 times with respect to control cells) in at least 4/10 immune challenges and in 4/6 times for each challenge, and with a maximal expression level of log_2_(f2/f1) < 0.6785 in only one point per challenge (a maximal expression level less than 1.6 times with respect to control cells in only one point). For *D. melanogaster*, gene definitions were as follows: **Immunity related genes**: genes associated with immunity based on Gene Onthology classification (GO:0006952, defense response, biological process), and with expression values in log_2_(f2/f1) > 0 in 6/6 time points of bacterial challenge. **Non-modified genes**: genes with an expression mean of log_2_(f2/f1) ± 0.1 and a standard deviation of ± 0.07 in 6/6 time points of bacterial challenge. **Down-regulated genes**: genes with an expression level of log_2_(f2/f1) ≤ -0.5855 (a repression level of at least 1.5 times with respect to control) in 6/6 times of the bacterial challenge. Two additional groups were included in the analysis: **Random genes**: two groups of 20 and 30 genes were randomly selected from the *A. gambiae *and *D. melanogaster *genomes, respectively, using the "Random Gene Selection" tool of RSA-Tools [65]. **Random sequences (artificial)**: two groups of 20 and 30 random non-biological sequences were generated using the "Random DNA sequence" tool of Sequence Manipulation Suite, version 2 [66], using this tool we generated random sequences with equal proportions of each nucleotide (~0.25).

Using these definitions, for *A. gambiae*, we first selected the expression profiles and the associated gene was then identified. To verify the annotated transcription initiation site, each gene prediction was manually curated by two approaches: The first was by aligning corresponding EST clusters obtained from AnoEST [67] and UNIGENE [68] to the *A. gambiae *genome (AgamP3, Ensembl release 45, Jun 2007) using BLAST in the ENSEMBL genome browser [28]. The second was based on manual verification of the presence of either TATA-box, Initiator sequence (Inr) or downstream promoter element (DPE) [69]. For *Drosophila*, the gene ID was included in the microarray database.

Once the gene associated with each profile was identified, the 5' regulatory regions were recovered for *D. melanogaster *(BDGP4.3) and *A. gambiae *(AgamP3, Ensembl release 45, Jun 2007) genes using Ensembl's data mining tool Biomart [70].

An additional set of 5' upstream 2000 bp sequences from the 12 *Drosophila *species derived from the 12 Drosophila genome project [32] retrieved from [71], release R1.1 for *D. virilis *(23 sequences), R1.2 for *D. ananassae *(23 sequences), *D. erecta *(27 sequences), *D. grimshawi *(21 sequences), *D. mojavensis *(21 sequences), *D. persimilis *(20 sequences), *D. sechellia *(28 sequences), *D. simulans *(29 sequences), *D. yakuba *(27 sequences) and *D. willistoni *(24 sequences), R2.2 for *D. pseudooscura *(27 sequences) and R5.7 for *D. melanogaster *(35 sequences) and 15 *Aedes aegypti *[72] 2500 bp sequences retrieved from Biomart [70] (AAEGL1) were also included for motif over-representation analysis. Selection of the *Drosophila *sequences was done based on orthology to *D. melanogaster *immunity gene data set described in Table 16, according to FlyBase annotations, but not expression data. For *Ae. aegypti*, genes were also selected based on one to one orthology to immunity genes in *A. gambiae *according to Table 17.

### Statistically overrepresented DNA motifs

To identify statistically overrepresented DNA motifs in 5' DNA regulatory regions of selected genes, we used the Oligo-Analysis program [29] searching for DNA motifs of 2 to 8 nucleotides of length in 5' upstream regions of 2500 or 2000 nucleotides. For the analysis, we created our own expected frequency tables for each motif length, using 5' upstream regions of 2500 nucleotides length corresponding to 13,172 genes of *D. melanogaster*; 13,166 genes of *A. gambiae*, and 16,691 in *Ae. aegypti*. A similar approach was used for the 11 additional *Drosophila *species using pre-computed 2000 bp upstream 5' sequences. The obtained expected frequency tables were used to estimate the expected number of occurrences for each oligonucleotide in induced, down-regulated, non-modified, random biological and random no-biological sets of sequences. The analyzed sequences were aligned to detect and avoid duplication between sequences, and duplicated regions larger than 40 nucleotides inside a sequence were removed. Also, to prevent a bias due to self-overlapping, a non-overlapping mode was adopted. The detection of overrepresented oligonucleotides was based on an estimation of the significance of the observed occurrences (O_occ_). For each oligonucleotide, the p value (P_occ_) was calculated on the basis of the binomial distribution. Because the analysis comprise multiple tests (256 in the case of tetranucleotides), the possibility exists that even low p values appeared by chance. To correct for such a multitesting effect, the p values were multiplied by the number of oligonucleotides. This correction results in an expected value (E_occ_). The significance index [sig_occ _= -log(E_occ_)] reflects the degree of overrepresentation for each oligonucleotide in a logarithmic scale [30].

The motifs overrepresentation identified with Oligo-Analysis, was verified using POBO [31], which uses bootstrap to verify the statistical overrepresentation of a given motif.

### Nucleosome positioning prediction

To predict regions of nucleosomal occupancy in the sequences of the distinct groups of *A. gambiae *and *D. melanogaster *5'-US, we used two programs: RECON [22], which assigns a nucleosomal potential value at each position in a sequence using sliding windows of 160 pb based on statistical distribution of dinucleotide frequencies, and Nucleosome Position Prediction [20], which predicts nucleosomal positioning using probabilistic and thermodynamic models, assigning a p value of nucleosomal occupancy (pNO) to each position of a sequence. For this last program, we analyzed 5'-US using yeast, chicken and human models, and both published and working versions of the program. The length of the 5'-US analyzed was of 2500 bp for *A. gambiae*, *D. melanogaster *and *Ae. aegypti *and 2000 bp for non-melanogaster *Drosophila *species. For RECON, we used 2660 bp that comprised the 2500 pb promoter, flanked by 80 bp, in order to recover nucleosomal potential values for all promoter positions.

### Analysis of motifs position regarding predicted nucleosomal regions

To associate the results obtained with the program Oligo-Analysis and those obtained with RECON and Nucleosome Position Prediction, perl scripts were written to automatically associate motifs coordinates (obtained with DNA-Pattern, [65] with tables containing "Nucleosomal potential" values (obtained with RECON) or "p of nucleosomal occupancy (pNO)" values (obtained with Nucleosome Position Prediction).

To determine if there was a correlation between the findings of Oligo-analysis and RECON, using perl scripts, the corresponding value of nucleosomal potential obtained with RECON was assigned to each position of the ATAA motif, having four values for each motif, on basis to these four values each ATAA motif was classified as: 1) positive, if the four positions of ATAA were positive; 2) negative, if the four positions were negative; 3) mixed, if at least one position was of opposite sign to the others, and 4) undefined, if at least one position was an N. Once classified, the distribution of ATAA motifs between these four categories was statistically evaluated.

Similarly, to determine if there was a correlation between the findings of Oligo-analysis and Nucleosome Position Prediction, using perl scripts, the corresponding pNO value obtained with the algorithm "Nucleosome Position Prediction" was assigned to each position of the ATAA motif, having again four values for each motif, one per each position. Based on these four values, each ATAA motif was classified as: 1) high p value motif if the four positions had occupancy p values higher than 0.8, 2) medium p value motif if the four positions had occupancy p values between 0.8 an 0.5, 3) low p value motif if the four positions had occupancy p values below of 0.5, and 4) undefined if at least one value were not belonging to the same range of values. Additionally, given that this software uses yeast, chicken and human models, and have a working and a published version, data generated with each model and each version were analyzed. This program does not accept sequences with Ns, therefore, in some cases the number of sequences analyzed by group of 5' upstream sequences was slightly smaller. For *A. gambiae*: 16 immunity, 12 down-regulated, 17 random genes and 17 non-modified. For *D. melanogaster*, only the group of non-modified genes was modified, from 32 to 31 sequences. For the other *Drosophila *species and *Ae. aegypti *the number of analysed sequences was the same for both programs.

### Analysis of 5'-US with alignment matrices

**Figure 12:**
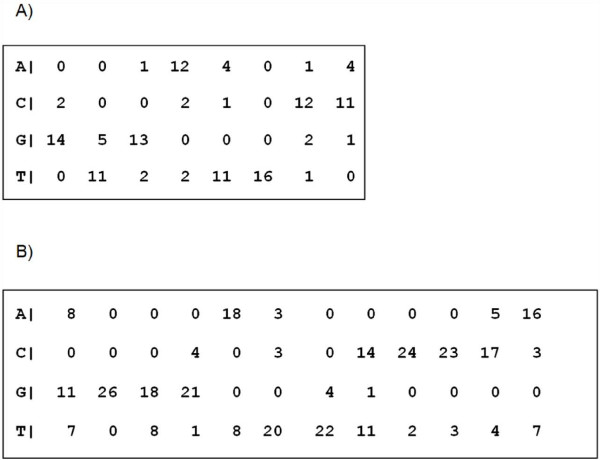
**Alignment matrices for NFκB motifs identified by MEME**[34]**in immunity genes of *A. gambiae *(A) and *D. melanogaster *(B).**

Using alignment matrices constructed on NFκB REs identified in immunity 5'-US of *A. gambiae *and *D. melanogaster *by MEME [35] (Figure 12), a matrix-based search was carried out in the different groups of genes using the PATSER algorithm [73], searching for NFκB REs. A lower threshold estimation of 5.0 was assigned.

### Statistical analysis

In order to compare the distribution of AT-rich motifs associated with positive nucleosomal potential values among groups of genes, we fitted ordinary least squares regression models with robust standard errors, with the number of positive AT-rich motifs as the dependent variable and dummy variables of the corresponding group of genes as predictors for all dipteran species. We fitted similar models to compare among groups of genes the distribution of AT-rich motifs associated with pNO > 0.8, and the AT% difference between groups of genes. Additionally, we compared the number of AT-rich motifs within all types of nucleotide sequences, either associated with positive vs. negative values of nucleosomal potential or pNO > 0.8 vs. pNO < 0.5, by use of paired Student's T tests.

To evaluate the distribution of NFκB REs throughout the promoter regions, comparisons were done using Poisson regression with the count of NFκB RE as response variable and the group of genes as independent variable. The models support the χ^2 ^goodness-of-fit test, when the model was not supported, a Kruskal-Wallis non-parametric test was done. Ordinary least squares regression analysis was performed to compare the counts of ATAA in the vicinity (± 200 bp) of NFκB motifs by type of gene group (immunity, non-modified and down-regulated), in both *A. gambiae *and *D. melanogaster*.

## Authors' contributions

JH–R participated in the design, data acquisition, data analysis and interpretation and wrote the manuscript. HS, FJC–R wrote perl scripts for data acquisition. HL–F performed statistical analysis. VV–G participated in data interpretation as well as revising the manuscript. MHR participated in conception, data analysis and interpretation as well as revising the manuscript. JM–B participated in conception and design, data acquisition and analysis, interpretation and drafted the manuscript.

## Supplementary Material

Additional file 1 Contains Table 18. 4 bp motif enrichment in 5'-US of Immunity genes of the *Drosophila *genus and *Aedes aegypti*. Click here for file
