# Supplementary material for: Immunity related genes in dipterans share common enrichment of AT-rich motifs in their 5' regulatory regions that are potentially involved in nucleosome formation
Source: BMC Genomics. 2008 Jul 9;9:326. doi: 10.1186/1471-2164-9-326 (PMC2491644; doi:10.1186/1471-2164-9-326)
Supplement: Additional file 1 — Contains Table 18. 4 bp motif enrichment in 5'-US of Immunity genes of the Drosophila genus and Aedes aegypti. [file 1471-2164-9-326-S1.pdf]

Table 18. 4 bp motif enrichment in 5'-US of Immunity genes of the *Drosophila* genus and *Aedes aegypti*.

D. melanogaster		
**Motif**	**occ_sig**	**rank**
**aaaa**	**15.8**	**1**
**ataa**	**11.59**	**2**
**tata**	**6.82**	**3**
**aaat**	**6.69**	**4**
**taaa**	**6.49**	**5**
**aata**	**5.75**	**6**
**aatt**	**4.61**	**7**
gaaa	4.04	8
**atat**	**3.97**	**9**
cccc	2.42	10
D. simulans		
**Motif**	**occ_sig**	**rank**
**aaaa**	**7.49**	**1**
**tata**	**6**	**2**
**ataa**	**4.87**	**3**
cccc	4.09	4
**taaa**	**3.71**	**5**
gtaa	3.06	6
aaag	2.95	7
**aatt**	**2.95**	**8**
aaga	2.82	9
**atat**	**2.41**	**10**
D. sechellia		
**Motif**	**occ_sig**	**rank**
aaag	3.05	1
gaaa	2.74	2
**taaa**	**2.34**	**3**
agat	1.72	4
gata	1.67	5
gtaa	1.48	6
**ataa**	**1.31**	**7**
tcaa	1.23	8
atac	1.11	9
**aaat**	**1.03**	**10**
D. yakuba		
**Motif**	**occ_sig**	**rank**

atag	2.79	1
gata	1.79	2
**tata**	**1.3**	**3**
**ataa**	**0.59**	**4**
aaag	0.55	5
atac	0.33	6
cata	0.3	7
**ttaa**	**0.18**	**8**
D. erecta		
**Motif**	**occ_sig**	**rank**
**tata**	**6.39**	**1**
**atat**	**4.42**	**2**
**aaaa**	**4.24**	**3**
cata	4.08	4
taca	3.1	5
atac	2.49	6
**ataa**	**2.46**	**7**
cccc	2.33	8
aaag	1.12	9
**taaa**	**0.75**	**10**
D. ananassae		
**Motif**	**occ_sig**	**rank**
**tata**	**1.83**	**1**
**ataa**	**1.3**	**2**
aatc	0.95	3
atca	0.7	4
atag	0.59	5
**aaat**	**0.46**	**6**
**ttaa**	**0.45**	**7**
atat	0.36	8
aaca	0.3	9
**aaaa**	**0.08**	**10**
D. pseudoobscura		
**Motif**	**occ_sig**	**rank**
**tata**	**2.92**	**1**
agag	2.08	2
caga	1.86	3
agat	1.82	4
aaag	1.62	5
agac	0.7	6
**ataa**	**0.15**	**7**
gaga	0.15	8
aaga	0.1	9

ccta	0.05	10
D. persimilis		
**Motif**	**occ_sig**	**rank**
caga	2.52	1
agat	1.51	2
cata	0.61	3
aaag	0.45	4
D. willistoni		
**Motif**	**occ_sig**	**rank**
**tata**	**5.01**	**1**
**atat**	**4.49**	**2**
gaaa	1.18	3
atac	1	4
taca	0.82	5
**aaaa**	**0.62**	**6**
aatt	0.58	7
acat	0.58	8
D. virilis		
**Motif**	**occ_sig**	**rank**
cccc	2.03	1
**aaaa**	**0.62**	**2**
ccga	0.11	3
D. mojavensis		
**Motif**	**occ_sig**	**rank**
**tata**	**2.87**	**1**
ggga	2.36	2
taga	1.91	3
agat	1.09	4
agtc	0.86	5
agac	0.78	6
cccc	0.47	7
taca	0.31	8
aaga	0.15	9
D. grimshawi		
**Motif**	**occ_sig**	**rank**
**tata**	**2.14**	**1**
agag	1.75	2
**atat**	**1.54**	**3**
**aaaa**	**1.14**	**4**
**ataa**	**0.94**	**5**
**aata**	**0.03**	**6**

Ae. aegypti		
**Motif**	**occ_sig**	**rank**
tcaa	4.27	1
**ttaa**	**3.36**	**2**
**aaat**	**3.32**	**3**
**aatt**	**2.78**	**4**
tgaa	1.43	5
**taaa**	**1.42**	**6**
caaa	0.91	7
**atat**	**0.36**	**8**
gtaa	0.16	9
catg	0.1	10

AT-rich motifs used to build Figure 4 are indicated in bold.
